# Vitamin D Deficiency, Obesity, and Metabolic Parameters in Chilean Older Adults

**DOI:** 10.3390/jpm16020090

**Published:** 2026-02-04

**Authors:** Mirelly Álamos, Bárbara Leyton, Alejandra Parada, Bárbara Angel

**Affiliations:** 1 Departamento de Nutrición y Dietética, Escuela de Ciencias de la Salud, Facultad de Medicina, Pontificia Universidad Católica de Chile, Santiago 7820436, Chile; malamos@uc.cl (M.Á.); acparada@uc.cl (A.P.); 2 Public Health Nutrition Unit, Institute of Nutrition and Food Technology, University of Chile, Santiago 7830490, Chile; bleyton@inta.uchile.cl; 3 Escuela de Obstetricia, Facultad de Ciencias para el Cuidado de la Salud, Universidad San Sebastián, Santiago 7510157, Chile

**Keywords:** vitamin D deficiency, older adults, obesity, metabolic parameters

## Abstract

**Background/Objectives**: Vitamin D deficiency and obesity are prevalent public health concerns among older adults, with potential impacts on metabolic health. Despite high deficiency rates reported globally, data on their relationship in Chilean older populations remain limited. This study investigates the relationship between 25(OH)D status, obesity, and metabolic parameters in Chilean older adults using data from the 2016–2017 National Health Survey (ENS). **Methods**: A cross-sectional analysis was conducted in 1252 individuals aged ≥ 65 years with complete 25(OH)D and anthropometric measurements. Plasma levels of 25(OH)D were classified as optimal ≥ 30 ng/mL, insufficiency 20–29.9 ng/mL, deficiency 12–19.9 ng/mL, and severe deficiency < 12 ng/mL. Logistic regression models adjusted for age, sex, education, comorbidities, and environmental factors were used to assess associations. **Results**: The results demonstrated that 88.3% of older adults had 25(OH)D ≤ 30 ng/mL, with 58.3% presenting deficiency. Obesity was an independent risk factor for vitamin D deficiency across all models. Geographic location, female sex, and smoking also influenced deficiency risk, while no significant associations emerged with type 2 diabetes or hypertension. **Conclusions**: These findings highlight the need for targeted strategies addressing vitamin D insufficiency in older adults, considering regional and lifestyle factors, to improve health outcomes in this vulnerable population.

## 1. Introduction

Vitamin D is a fat-soluble vitamin existing in two distinct forms: ergocalciferol (D2) found in yeast and plant-based foods, and cholecalciferol (D3) present in fatty fish, eggs, fortified dairy products, and supplements [1]. However, the primary source of vitamin D3, accounting for 80–90% of total body stores, is its synthesis in the skin via ultraviolet (UV) radiation-induced conversion of 7-dehydrocholesterol [1,2].

The metabolic pathway of vitamin D involves sequential hydroxylation: vitamin D metabolites (D2 and D3) undergo 25-hydroxylation in the liver via 25-hydroxylase to produce 25(OH)D, followed by 1-alpha-hydroxylation in the kidneys to form 1,25α(OH)2D (calcitriol), the biologically active form [2]. Local vitamin D production can occur in multiple tissues, generating auto- and paracrine effects [2]. Observational studies have reported that lower serum 25(OH)D concentrations are associated with higher blood pressure and an increased risk of hypertension, possibly through effects on the renin–angiotensin–aldosterone system; however, this relationship remains non-causal and should be interpreted with caution [3,4]. Similarly, type 2 diabetes mellitus (DM2) patients demonstrate lower 25(OH)D concentrations compared to non-diabetic populations, with inverse associations observed between vitamin D status and fasting glucose, impaired glucose tolerance, and HbA1c levels [5,6]. 25(OH)D, which has an approximate half-life of 3 weeks, represents the principal biomarker for assessing vitamin D status in clinical practice, in contrast to the active metabolite 1,25α(OH)2D, which has a half-life of only 4–6 h [2,7].

The Endocrine Society of the United States established guidelines defining vitamin D status as follows: deficiency when 25(OH)D < 20 ng/mL, insufficiency between 20–29 ng/mL, sufficiency ≥ 30 ng/mL for optimal musculoskeletal health, and severe deficiency < 12 ng/mL [8]. Using the <20 ng/mL threshold, approximately one-third of the global population presents vitamin D deficiency, while severe deficiency affects approximately 7% worldwide with considerable variation across countries and populations [9,10,11].

Older adults are particularly vulnerable to 25(OH)D deficiency due to inadequate dietary intake and limited sun exposure [11,12]. Aging further diminishes 7-dehydrocholesterol concentration in the epidermis and total previtamin D3 production following sun exposure [13,14]. Previous Chilean research has documented elevated prevalence of vitamin D deficiency in older adults, with fewer than 10% achieving sufficient levels, and deficiency occurring in 80% of hip fracture patients [15]. Other studies report deficiency prevalence ranging from 36.5% to 70% in Chilean older populations [16,17].

The bidirectional relationship between vitamin D status and chronic non-communicable diseases has been extensively investigated [7,18,19,20,21,22,23]. The inverse relationship between vitamin D insufficiency and obesity has garnered substantial research attention over the past decade [19,21,24,25,26,27,28,29,30]. Both conditions represent modifiable risk factors for cardiovascular disease, with serum 25(OH)D concentrations inversely related to multiple cardiovascular risk factors including obesity and glucose metabolism alterations [27,31,32,33].

Proposed mechanisms for explaining vitamin D deficiency in obesity include (1) reduced sun exposure among obese individuals [34], (2) decreased bioavailability of dietary vitamin D due to sequestration in adipose tissue [25], and (3) reduced expression of key hydroxylating enzymes (CYP2R1, CYP27B1) in subcutaneous adipose tissue of obese individuals, resulting in lower concentrations of active vitamin D metabolites [34].

The 2016–2017 National Health Survey (ENS) represented Chile’s first population-level vitamin D assessment, incorporating 25(OH)D measurement as an innovation in epidemiological surveillance [35]. Limited Chilean research has explored the relationship between obesity and vitamin D deficiency in older adults, with conflicting results. The ALEXANDROS cohort identified a significant interaction between 25(OH)D deficiency (<20 ng/mL) and abdominal obesity in 1180 community-dwelling older adults (*p* < 0.010) [17], whereas a separate 453-person cohort of healthy Chilean older adults (≥60 years) found no significant obesity–vitamin D association [15].

Meta-analytical evidence reveals inconsistent obesity–vitamin D associations across populations. A meta-analysis examining the correlation between serum 25(OH)D and body mass index (BMI) in developed and developing nations demonstrated weak overall correlations [36]. Additional meta-analytical evidence suggests that individuals with overweight and obesity across different age groups present similar probabilities of vitamin D deficiency, indicating that age may not substantially contribute to this association [37].

Given the elevated prevalence of vitamin D deficiency in Chilean older adults and the contradictory findings regarding its association with obesity, the present study aims to investigate the relationship between 25(OH)D levels, obesity, and metabolic parameters in Chilean older adults using data from the 2016–2017 National Health Survey (ENS). Understanding the individualized risk profiles of vitamin D deficiency in this population has direct implications for developing preventive interventions aimed at older adults at higher risk.

## 2. Materials and Methods

This cross-sectional, analytical, retrospective study utilized secondary data analysis from the 2016–2017 National Health Survey of Chile (ENS). The ENS is a nationally representative, multi-stage, stratified random cluster sampling survey conducted across urban and rural areas of Chile’s 15 administrative regions. The survey included 6233 participants aged ≥ 15 years, both Chilean citizens and foreign residents residing in occupied private dwellings. The sampling methodology involved stratification into 30 strata representing urban and rural areas, with counties designated as primary sampling units, followed by households, and finally individual selection within households.

Survey weights accounted for differential selection probabilities and non-response rates, with post-stratification allowing sample extrapolation to the estimated Chilean population. Interviews were conducted in participants’ homes using electronic data capture, administered by trained interviewers and nurses according to questionnaire type [35].

The 2016–2017 ENS protocol and written informed consent received ethical approval from the Scientific Ethics Committee of the Faculty of Medicine at the Pontificia Universidad Católica de Chile, Santiago, Chile (CEC-MedUC, project number 16-019) and adhered to the Declaration of Helsinki. This study received a waiver of informed consent from the Institute of Nutrition and Food Technology ethical–scientific committee, University of Chile, Santiago, Chile.

Participants meeting the following criteria were included: Age ≥ 65 years, complete data for BMI (kg/m^2^), complete 25(OH)D measurement data.

### 2.1. Variables and Definitions

The primary outcomes assessed included vitamin D status, obesity, type 2 diabetes mellitus (DM2), hypertension (HT), and metabolic syndrome (MS). Vitamin D status followed Endocrine Society criteria, categorizing serum 25(OH)D concentrations as optimal/sufficient (≥30 ng/mL), insufficient (20–29.9 ng/mL), deficient (12–19.9 ng/mL), or severely deficient (<12 ng/mL); or as a dichotomous variable, deficiency ≤ 20 ng/mL and sufficiency > 20 ng/mL. Fasting blood samples (5 mL) were collected primarily during spring and summer months (September 2016–February 2017), with serum 25(OH)D quantified via liquid chromatography–tandem mass spectrometry (LC-MS/MS), the reference standard method separately measuring 25-OH-D3, 25-OH-D2, EPI-25-OH-D3, and EPI-25-OH-D2.

The body mass index (BMI: Weight/Height^2^ = kg/m^2^) was calculated and nutritional status was determined using the BMI categories according to WHO Cut-off points.

Abdominal obesity utilized waist circumference measured at the midpoint between the iliac crest and costal margin (WHO method), applying cutoffs of ≥90 cm for men and ≥80 cm for women. Type 2 diabetes mellitus diagnosis required fasting plasma glucose ≥ 126 mg/dL (≥8 h fasting) and/or self-reported physician diagnosis (excluding gestational diabetes) per American Diabetes Association criteria, with in-home capillary glucose (Hemoglucotest) and venous blood sampling for laboratory glucose and HbA1c determination.

Metabolic syndrome was diagnosed when participants met ≥ 3 of the following criteria: abdominal obesity, elevated blood pressure, elevated triglycerides, low HDL cholesterol, or fasting glucose ≥ 100 mg/dL. Hypertension was established by mean arterial pressure from three measurements ≥ 140/90 mmHg and/or self-reported antihypertensive pharmacological treatment. Tobacco use was assessed by self-reported consumption and was dichotomously classified into two categories: current smoker, defined as smoking ≥ 1 cigarette daily, and non-smoker, comprising those who reported no current tobacco use.

Physical activity levels were evaluated using the Global Physical Activity Questionnaire (GPAQ) and stratified as low, moderate, or high. Educational attainment reflected self-reported total years of schooling, grouped as low (<8 years), medium (8–12 years), or high (>12 years). Solar exposure was self-perceived via the question “Over the past week, how much sunlight have you been exposed to?” (much vs. little), and fish consumption frequency included categories of once weekly, <3 times monthly, <once monthly, or never. Geographic location employed five Chilean macrozone classifications: North (Tarapacá, Antofagasta, Arica y Parinacota), Center (Coquimbo, Valparaíso, Metropolitan Region), Center–South (Libertador Bernardo O’Higgins, Maule, Biobío), South (La Araucanía, Los Ríos, Los Lagos), and Austral (Aysén, Magallanes y Antártica). In addition, age was categorized into three groups (65–74, 75–84, and ≥85 years), chosen to reflect functional and epidemiological differences in older adults; these age groups were entered as categorical predictors in the regression models, using 65–74 years as the reference category.

Covariables included sex, educational attainment, physical activity, tobacco use, alcohol consumption, fish consumption, age groups, and geographic macrozone, selected to comprehensively adjust for demographic, behavioral, and environmental confounders in multivariate analyses.

### 2.2. Statistical Analysis

Descriptive analyses presented continuous variables as mean ± standard deviation (SD) with 95% confidence intervals (95% CI). Categorical variables were reported as absolute frequencies and percentages (%).

For normally distributed data with homogeneous variance, independent samples *t*-tests compared continuous variables between groups; chi-square tests compared categorical variables while two-sample proportion tests were used for comparing the prevalence. Logistic regression analysis assessed associations between 25(OH)D deficiency (dependent variable) and obesity (independent variable).

Three progressive models were constructed:

- Model 1: Adjusted for sex, educational attainment, and age groups.
- Model 2: Model 1 adjustments plus DM2, HT, and Physical Activity (GPAQ).
- Model 3: Model 1 and 2 adjustments plus geographic location (Macrozone), sun exposure, fish consumption, and tobacco use.

Vitamin D-25(OH)D associations with DM2 were similarly analyzed with equivalent adjustment models in separate logistic regression models (Supplementary Table S1). Survey expansion factors were incorporated into all analyses. Statistical significance was set at *p* < 0.05. All analyses were performed using STATA 16 software (StataCorp, College Station, TX, USA).

## 3. Results

### 3.1. Population Characteristics

Of the 6233 initial ENS 2016–2017 participants, 1252 older adults (≥65 years) possessed complete 25(OH)D and anthropometric data, representing 1,754,379 individuals in the expanded national sample (Table 1). 43.6% of the older adults included in this study are men, representing 766,084 people in the expanded sample, while women account for 988,295 people, which corresponds to 56.3%. Mean age was 74.2 ± 6.9 years in women and 73.8 ± 6.8 years in men without significant between-sex differences (*p* = 0.312).

The age distribution showed a decreasing number of participants with advancing age. The majority of participants resided in urban areas (80.2%), while 19.8% lived in rural areas. Women had lower educational attainment than men, with 61.5% of women having less than 8 years of education compared to a lower proportion among men. More than half of older adults (54.5%) were classified as overweight or obese, with obesity alone affecting 24.5% of the sample (29.3% of women vs. 16.0% of men, *p* < 0.0001).

Physical activity assessment via the Global Physical Activity Questionnaire (GPAQ) revealed significant sex differences, with 55.6% of men achieving high activity levels compared to lower proportions in women. Data on vitamin D supplementation or dietary vitamin D intake were unavailable in the ENS dataset.

### 3.2. Comorbidity Prevalence

Hypertension, diagnosed by blood pressure ≥ 140/90 mmHg or antihypertensive medication use, affected 72.8% of the people without significant sex differences. Metabolic syndrome prevalence was 62.4% among 862 participants with complete diagnostic data. Abdominal obesity, defined as waist circumference ≥ 80 cm in women and ≥90 cm in men, was present in 84.6% of the total sample, with a significantly higher prevalence among women (89.9%) compared to men (75.1%, *p* < 0.001). Type 2 diabetes mellitus was documented in 14.4% of the cohort without significant sex-based differences (women 14.1%, men 14.9%, *p* = 0.67).

### 3.3. Biochemical and Anthropometric Parameters

Serum 25(OH)D concentrations were significantly lower in women (mean 17.9 ± 8.6 ng/mL) than in men (mean 21.1 ± 8.7 ng/mL, *p* < 0.001) (Table 2). In addition, women had significantly higher serum total cholesterol (186.4 ± 41.9 mg/dL vs. 174.3 ± 39.6 mg/dL in men, *p* < 0.001) and HDL cholesterol (51.9 ± 14.1 mg/dL vs. 44.4 ± 12.3 mg/dL in men, *p* < 0.001). Mean BMI was significantly elevated in women (29.4 ± 5.4 kg/m^2^) versus men (27.8 ± 4.2 kg/m^2^, *p* < 0.001). Mean waist circumference was 95.5 ± 13.2 cm in women versus 97.7 ± 12.9 cm in men (*p* = 0.004). No significant differences were found for triglycerides, fasting glucose, or HbA1c.

**Figure 1:**
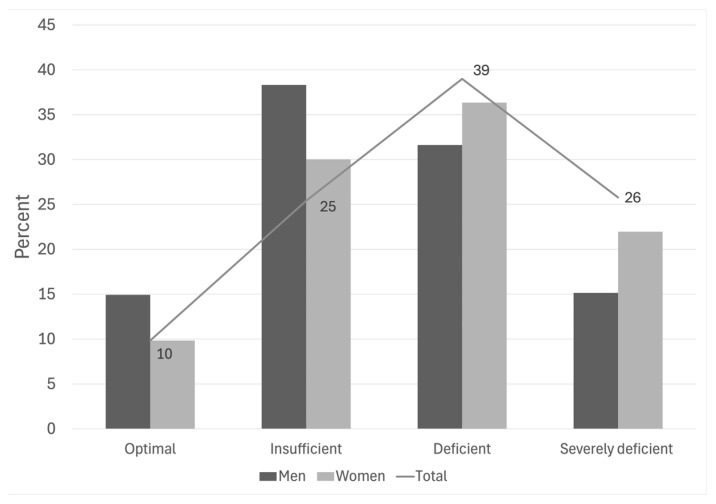
Distribution of 25(OH)D categories by sex in Chilean older adults. The graphic showing the percentage of Chilean older adults with optimal (≥30 ng/mL), insufficient (20–29.9 ng/mL), deficient (12–19.9 ng/mL), and severely deficient (<12 ng/mL) serum 25(OH)D concentrations, stratified by sex; the line represents the total prevalence for each category.

Over 88% of older adults presented 25(OH)D ≤ 30 ng/mL. Only 11.6% achieved optimal vitamin D status (≥30 ng/mL), while 30.0% had insufficiency (21–29 ng/mL), 36.3% presented deficiency (12–20 ng/mL), and 21.9% demonstrated severe deficiency (<12 ng/mL). Significant sex differences were evident, with women showing higher deficiency (38.9%) and severe deficiency (25.7%) prevalence compared to men (31.6% and 15.4%, respectively, *p* < 0.0001) Figure 1.

Age group analysis demonstrated that optimal vitamin D status was concentrated primarily in the 75–84 years age group with predominantly male representation. Severe deficiency prevalence increased with advancing age, exceeding 36% in participants ≥ 85 years. Age group and vitamin D status showed a significant association (*p* = 0.029).

### 3.4. Associations Between Vitamin D Status, Nutritional Status, and Comorbidities

Logistic regression analysis examined the independent association between vitamin D deficiency (25(OH)D ≤ 20 ng/mL) and obesity, with progressive adjustment for confounding variables (Table 3). In Model 1, which adjusted for sex, educational attainment, and age groups, obesity emerged as an independent risk factor for vitamin D deficiency (OR = 1.80, 95% CI 1.31–2.48, *p* < 0.001). This association persisted in Model 2, which additionally adjusted for hypertension, DM2, and physical activity (OR = 1.57, 95% CI 1.13–2.19, *p* = 0.01), and remained significant in Model 3 after further adjustment for geographic macrozones, solar exposure, fish consumption, and tobacco use (OR = 1.55, 95% CI 1.10–2.18, *p* = 0.01).

Female sex consistently emerged as a significant predictor of vitamin D deficiency across all models (Model 1: OR = 1.94, *p* < 0.001; Model 2: OR = 1.81, *p* < 0.001; Model 3: OR = 1.66, *p* < 0.001). Geographic location showed marked associations in Model 3, with increased deficiency risk in the Center (OR = 2.96, *p* < 0.001), Center–South (OR = 2.01, *p* < 0.001), South (OR = 2.16, *p* < 0.001), and Austral (OR = 4.73, *p* < 0.001) regions compared to the North. Current smoking status increased deficiency risk (OR = 1.66, 95% CI 1.10–2.51, *p* = 0.02 in Model 3).

Analysis of 25(OH)D associations with DM2 revealed no significant independent relationships in any regression model after adjusting for confounding variables and the OR is reported in the Supplementary Material.

## 4. Discussion

The present study documents markedly elevated vitamin D deficiency prevalence in Chilean older adults, with 88.1% presenting 25(OH)D ≤ 30 ng/mL and 58.3% meeting deficiency criteria (≤20 ng/mL). This prevalence substantially exceeds that observed in some international cohorts and aligns with previous smaller Chilean studies reporting deficiency in 36.5% to 70% of older populations [16,17]. The particularly high prevalence in this nationally representative sample highlights vitamin D insufficiency as a major public health concern in the Chilean aging population.

Sex-stratified analysis revealed significantly higher vitamin D deficiency in women (66.1%) compared to men (47.6%), mirroring findings from international literature. Female predominance of vitamin D deficiency has been attributed to reduced sun exposure, lower dietary intake, and biological differences in vitamin D metabolism and storage [6,29].

Obesity emerged as an independent vitamin D deficiency risk factor across progressive regression models, with adjusted odds ratios remaining significant (Model 3: OR = 1.55, *p* = 0.01). This finding aligns with substantial literature documenting bidirectional associations between obesity and vitamin D insufficiency [6,19,20,21,24,25,26,28,36,37,38]. The persistence of this association across adjustment models suggests obesity represents a modifiable risk factor amenable to intervention targeting vitamin D status improvement.

Multiple mechanisms likely explain the obesity–vitamin D association observed in this cohort. First, obese individuals may exhibit reduced sun exposure, limiting cutaneous vitamin D synthesis [18,37]. Second, the lipophilic nature of vitamin D promotes sequestration in adipose tissue compartments, reducing bioavailability of both dietary and cutaneous sources [25]. Third, reduced expression of key hydroxylating enzymes (CYP2R1, CYP27B1) in adipose tissue of obese individuals decreases conversion of vitamin D precursors to active metabolites [34].

Geographic macrozone emerged as a strong vitamin D deficiency predictor, with southern regions showing substantially elevated risk (Austral: OR = 4.73). This finding reflects the inverse correlation between latitude and UVB radiation intensity in the Southern Hemisphere, with southern Chilean regions receiving substantially reduced ambient ultraviolet exposure particularly during winter months. This observation emphasizes that intervention strategies addressing vitamin D insufficiency in older adults must account for geographic variation in solar accessibility, particularly in populations residing at elevated southern latitudes.

Contrary to international observational data linking vitamin D insufficiency with DM2 and HT [3,4,5,6,20,39], logistic regression analyses revealed no significant associations in this cohort. Several explanations merit consideration: (1) the exceptionally high prevalence of vitamin D deficiency in this population may approach saturation, obscuring graded dose–response relationships; (2) disease prevalence rates may insufficiently discriminate between deficient and sufficient vitamin D categories in this aged group; (3) unmeasured confounding variables including vitamin D supplementation, dietary calcium, parathyroid hormone levels, or genetic polymorphisms affecting vitamin D metabolism may mask or modify true associations; (4) cross-sectional design precludes causal inference; and (5) temporal latency between vitamin D exposure and chronic disease manifestation may exceed the cross-sectional assessment window.

The identification of associations between obesity, geographic location, and vitamin D deficiency in Chilean older adults supports further investigation into personalized approaches to prevention and intervention. Obese older adults and those residing in southern latitudes may be at higher risk of 25(OH)D deficiency. Potential evidence-based interventions include dietary vitamin D counseling that emphasizes fish consumption, even though the present analysis did not demonstrate a significant association with vitamin D status, together with cautious promotion of sun exposure during appropriate hours. Additional strategies comprise vitamin D supplementation tailored to baseline 25(OH)D concentrations and individual risk profiles, as well as obesity reduction through structured physical activity and dietary interventions, which may improve vitamin D status through multiple mechanisms.

The finding that physical activity level did not significantly modify associations with vitamin D deficiency suggests that, while promoting activity is beneficial for overall health, additional vitamin D-specific interventions may be necessary in this population.

### Strengths and Limitations

The strengths of this investigation include the use of a nationally representative sample obtained through multi-stage stratified random sampling and the application of standardized LC-MS/MS methodology for 25(OH)D quantification, considered the reference standard. In addition, the study incorporated a comprehensive covariate assessment encompassing comorbidities, physical activity, dietary behaviors, and environmental exposures, and employed progressive regression models to statistically adjust for multiple potential confounding variables. Finally, it examined clinically relevant outcomes in an understudied population, thereby contributing novel evidence to the field.

The limitations of this study include its cross-sectional design, which precludes causal inference, and the lack of information on vitamin D supplementation and dietary vitamin D intake. In addition, it was not possible to assess bioavailable 25(OH)D or free vitamin D, which may better capture biologically active pools, and parathyroid hormone levels were not measured, limiting mechanistic interpretation. The single time-point assessment also prevented characterization of temporal relationships, and there is a possibility of residual confounding from unmeasured factors such as genetic polymorphisms, medication use, or other behavioral characteristics.

## 5. Conclusions

Chilean older adults exhibit exceptionally elevated vitamin D deficiency prevalence, with 88% presenting 25(OH)D ≤ 30 ng/mL. Obesity and female sex emerge as robust, independent risk factors for vitamin D deficiency, while geographic location (southern residence) substantially amplifies deficiency risk. These findings underscore the necessity for personalized, geographically-stratified, obesity-targeted prevention strategies addressing vitamin D insufficiency in this vulnerable population. Future research should employ prospective designs to establish causal relationships between vitamin D status and chronic disease outcomes, investigate mechanisms underlying the obesity–vitamin D association, and evaluate intervention efficacy in reducing deficiency prevalence and improving health outcomes in Chilean older adults. Integration of genomic and metabolomic approaches may refine individual risk prediction and personalization of vitamin D management strategies.

## Figures and Tables

**Table 1:** Demographic and Anthropometric Characteristics of Study Sample by Sex.

Characteristics	Men (43.6%)	Women (56.3%)	Total (n = 1252)	
	Mean ± SD	Mean ± SD	Mean ± SD	*p*
**Age (years)**	73.8 ± 6.8 **n %**	74.2 ± 6.9 **n %**	74.0 ± 6.8 **n %**	0.312 *
**Age Group (years)** 65–74 75–84 ≥85	272 60.6 145 32.3 32 7.1	470 58.5 249 34.7 54 6.7	742 59.3 424 33.9 86 6.9	0.676
**Geographic Area** Urban Rural	353 78.4 97 21.6	652 81.2 151 18.8	1004 80.2 248 19.8	0.233
**Educational Attainment (years)** <8 8–12 ≥13	234 52.5 154 34.5 58 13.0	488 61.5 251 31.6 55 6.9	722 58.2 405 32.7 113 9.1	<0.0001
**Nutritional Status** Normal Low weight Overweight Obesity	173 38.5 57 12.7 147 32.7 72 16.0	265 33.0 74 9.2 229 28.5 235 29.3	438 35.0 131 10.5 376 30.0 307 24.5	<0.0001

* Paired Student’s *t*-test mean and Pearson’s chi-squared test.

**Table 2:** Biochemical and Anthropometric Parameters by Sex.

Variable	Men Mean ± SD	Women Mean ± SD	Total Mean ± SD	*p* *
25(OH)D (ng/mL)	21.1 ± 8.7	17.9 ± 8.6	19.0 ± 8.8	<0.001
Waist circumference (cm)	97.7 ± 12.9	95.5 ± 13.2	96.3 ± 13.1	0.004
Total cholesterol (mg/dL)	174.3 ± 39.6	186.4 ± 41.9	180.4 ± 41.0	<0.001
HDL cholesterol (mg/dL)	44.4 ± 12.3	51.9 ± 14.1	49.4 ± 14.0	<0.001
LDL cholesterol (mg/dL)	101.4 ± 33.9	104.8 ± 35.6	103.7 ± 35.2	0.155
Triglycerides (mg/dL)	141.8 ± 77.0	147.4 ± 81.8	145.5 ± 80.2	0.303
Fasting glucose (mg/dL)	109.4 ± 43.2	108.4 ± 44.0	108.8 ± 43.7	0.677
HbA1c (g/dL)	6.6 ± 1.5	6.8 ± 1.8	6.7 ± 1.7	0.264
BMI (kg/m^2^)	27.8 ± 4.2	29.4 ± 5.4	28.8 ± 5.0	<0.001

* Paired Student’s *t*-test mean.

**Table 3:** Logistic Regression Analysis: Independent Associations of Vitamin D Deficiency (25(OH)D ≤ 20 ng/mL) with Obesity and Other Variables.

Vitamin D Deficiency (25(OH)D ≤ 20 ng/mL)	Model 1 OR (95%CI) *p*	Model 2 OR (95%CI) *p*	Model 3 OR (95%CI) *p*
Nutritional Status Low weight Overweight Obesity	1.09 (0.73–1.62) 0.69 1.14 (0.86–1.52) 0.36 1.80 (1.31–2.48) < 0.001	1.20 (0.79–1.84) 0.39 1.10 (0.82–1.48) 0.52 1.57 (1.13–2.19) 0.01	1.18 (0.76–1.84) 0.46 1.09 (0.81–1.49) 0.56 1.55 (1.10–2.18) 0.01
Female sex	1.94 (0.53–2.47) < 0.001	1.81 (1.41–2.33) < 0.001	1.66 (1.27–2.16) < 0.001
Educational Attainment 8–12 years >13 years	1.18 (0.91–1.53) 0.20 0.72 (0.47–1.08) 0.11	1.18 (0.90–1.55) 0.22 0.66 (0.43–1.02) 0.06	1.20 (0.91–1.59) 0.20 0.59 (0.37–0.92) 0.02
Age Groups 75–84 years >85 years	0.93 (0.72–1.20) 0.60 1.39 (0.85–2.26) 0.19	0.87 (0.66–1.14) 0.30 0.69 (1.93–0.59) 0.59	0.87 (0.66–1.15) 0.32 1.21 (0.71–2.06) 0.48
Comorbidities DM 2 Hypertension		1.23 (0.87–1.75) 0.24 1.12 (0.85–1.47) 0.42	1.38 (0.96–1.98) 0.08 1.09 (0.82–1.45) 0.56
Physical Activity (GPAQ) Moderate level High level		0.78 (0.58–1.06) 0.11 0.75 (0.55–1.01) 0.06	0.76 (0.56–1.04) 0.09 0.73 (0.53–1.01) 0.06
Geographic Macrozone Center Center-South South Austral			2.96 (2.03–4.32) < 0.001 2.01 (1.36–2.97) < 0.001 2.16 (1.41–3.31) < 0.001 4.73 (2.81–7.95) < 0.001
High Solar Exposure			0.77 (0.58–1.01) 0.06
Fish Consumption Once weekly ≤3 times monthly Once monthly or never			0.94 (0.58–1.52) 0.79 0.73 (0.44–1.21) 0.23 0.74 (0.45–1.19) 0.21
Current smoking			1.66 (1.10–2.51) 0.02

Model 1 adjusted for sex, educational attainment, and age groups. Model 2 additionally adjusted for hypertension, type 2 diabetes mellitus, and physical activity (GPAQ). Model 3 additionally adjusted for geographic macrozones, solar exposure, fish consumption, and tobacco use. Results presented as odds ratios (OR) with 95% confidence intervals (CI).

## Data Availability

The data from the ENS 2016–2017 are available at https://epi.minsal.cl/encuesta-ens/, accessed on 25 January 2026.

## Supplementary Materials

The following supporting information can be downloaded at: https://www.mdpi.com/article/10.3390/jpm16020090/s1, Figure S1: Histogram of serum 25(OH)D concentrations in Chilean older adults; Table S1: Logistic Regression Analysis: Independent Associations of Vitamin D Deficiency (25(OH)D ≤ 20 ng/mL) with DM2 and Other Variables.

## Abbreviations

The following abbreviations are used in this manuscript:

ENS	National Health Survey
OR	Odds Ratio
D3	cholecalciferol
DM2	Type 2 diabetes mellitus
BMI	Body mass index
HT	Hypertension
MS	Metabolic syndrome
LC-MS/MS	liquid chromatography–tandem mass spectrometry
WHO	World Health Organization
HbA1c	glycosylated hemoglobin
GPAQ	Global Physical Activity Questionnaire
