# Supplementary material for: Vitamin D Deficiency, Obesity, and Metabolic Parameters in Chilean Older Adults
Source: J Pers Med. 2026 Feb 4;16(2):90. doi: 10.3390/jpm16020090 (PMC12941998; doi:10.3390/jpm16020090)
Supplement: Supplementary file 1 [file jpm-16-00090-s001.zip › jpm-4105916-supplementary.pdf]

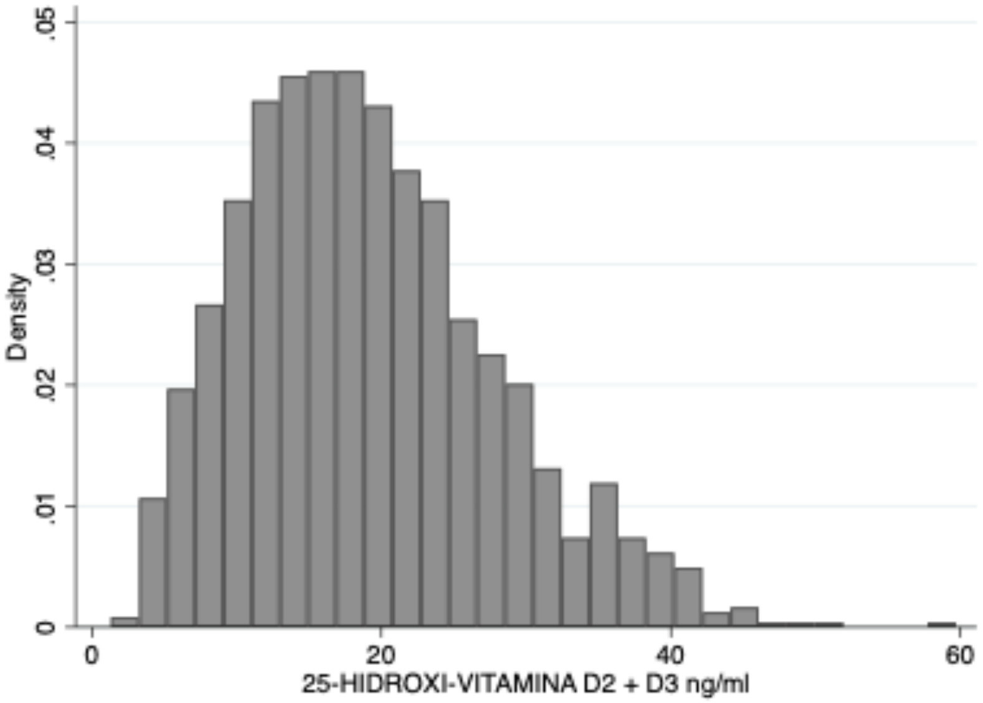


Supplementary figure 1: Histogram of serum 25(OH)D concentrations in Chilean older adults

**Supplementary Table 1.** Logistic Regression Analysis: Independent Associations of Vitamin D Deficiency (25(OH)D ≤20 ng/mL) with DM2 and Other Variables.

| Vitamin D Deficiency (25(OH)D ≤20 ng/mL) | **Model 1** **OR (95%CI) p** | **Model 2** **OR 95%CI p** |
|---|---|---|
| Diabetes Mellitus type 2 (presence) | 1.29 (0.92-1.82) 0.14 | 1.28 (0.90-1.80) 0.17 |
| Female sex | 2.03 (1.60-2.59) <0.001 | 1.81 (1.41-2.33) <0.001 |
| Educational Attainment | | |
| 8–12 years | 1.14 (0.88-1.47) 0.32 | 1.18 (0.90-1.55) 0.22 |
| >13 years | 0.65 (0.43-0.98) 0.04 | 0.66 (0.43-1.02) 0.06 |
| Age Groups | | |
| 75–84 years | 0.85 (0.65-1.10) 0.22 | 0.87 (0.66-1.14) 0.30 |
| >85 years | 1.11 (0.67-1.84) 0.68 | 0.69 (1.93-0.59) 0.59 |
| Physical Activity (GPAQ) | | |
| Moderate level | | 0.77 (0.57-1.04) 0.08 |
| High level | | 0.72 (0.53-0.97) 0.03 |

| | |
|---|---|
| Geographic Macrozone | |
| Center | 2.98 (2.05-4.33) <0.001 |
| Center-South | 2.04 (1.39-3.02) <0.001 |
| South | 2.28 (1.50-3.49) <0.001 |
| Austral | 5.03 (3.00-8.42) <0.001 |
| High solar exposure | 0.78 (0.59-1.02) 0.07 |
| Fish Consumption | |
| Once weekly | 0.93 (0.57-1.51) 0.77 |
| ≤ 3 times monthly | 0.75 (0.45-1.24) 0.26 |
| Once monthly or never | 0.74 (0.46-1.19) 0.22 |
| Current smoking | 1.58 (1.05-2.38) 0.03 |

Model 1 adjusted for sex, educational attainment, and age groups. Model 2 additionally adjusted Physical Activity (GPAQ), geographic macrozones, solar exposure, fish consumption and tobacco use. Results presented as odds ratios (OR) with 95% confidence intervals (CI).
